# How can we improve targeting of frail elderly patients to a geriatric day-hospital rehabilitation program?

**DOI:** 10.1186/1471-2318-10-82

**Published:** 2010-11-03

**Authors:** Silvia RM Pereira, Wendy Chiu, Alyson Turner, Stephanie Chevalier, Lawrence Joseph, Allen R Huang, Jose A Morais

**Affiliations:** 1 Academic Hospital São Francisco de Assis - Federal University of Rio de Janeiro, Avenida Presidente Vargas, 2863 Cidade Nova, Rio de Janeiro 22250-040, Brazil; 2 Division of Geriatric Medicine, McGill University Health Centre, Royal Victoria Hospital, 687 Pine Avenue West, Room M8.12, Montreal, Quebec, H3A 1A1, Canada; 3 Division of Clinical Epidemiology, McGill University Health Center, Royal Victoria Hospital, 687 Pine Avenue West, Room R4.27, Montreal, Quebec, H3A 1A1, Canada

## Abstract

**Background:**

The optimal patient selection of frail elderly persons undergoing rehabilitation in Geriatric Day Hospital (GDH) programs remains uncertain. This study was done to identify potential predictors of rehabilitation outcomes for these patients.

**Methods:**

This study is a retrospective cohort analysis of patients admitted to the rehabilitation program of our GDH, in Montreal, Canada, over a five year period. The measures considered were: Barthel Index, Older Americans Resources and Services, Folstein Mini Mental Status Exam, Timed Up & Go (TUG), 6-minute walk test (6 MWT), Gait speed, Berg Balance, grip strength and the European Quality of life - 5 Dimensions. Successful improvement with rehabilitation was defined as improvement in three or more tests of physical function. Logistic regression analysis using the Bayesian Information Criterion (BIC) was employed to select the optimal model for making predictions of rehabilitation success.

**Results:**

A total of 335 patients were studied, but only 233 patients had a complete data set suitable for the predictive model. Average age was 81 years and patients attended the GDH an average of 24 visits. Significant changes were found in several measures of physical performance for many patients ranging from improved gait speed in 21.3% to improved TUG in 62.7% of the cohort. Fifty-eight percent of patients attained successful improvement with rehabilitation by our criteria. This group was characterized by lower test scores on admission. Using BIC, the best predictor model was the 6 MWT [OR: 0.994 per meter walked (95% CI: 0.990-0.997)].

**Conclusions:**

The GDH rehabilitation program is effective in improving patients' physical performance. Although no single measure was found to be sufficiently predictive to help target candidates appropriately, the 6 MWT showed a trend to significance. Further research will be done to elucidate the utility of a composite 'rehab appropriateness index' and the role of International Classification of Function concepts for targeting frail elderly to GDH rehabilitation services.

## Background

Chronic illness and limitations in reserve capacity are more common in later life. Older people are more likely to experience sudden-onset conditions that impact on functional abilities, quality of life and mortality [1]. To meet the healthcare demands of an aging population, a spectrum of services and solutions have to be considered. Some authors have suggested that a Geriatric Day Hospital (GDH) rehabilitation program can prevent inappropriate acute admissions, facilitate earlier discharge from hospital, improve clinical symptoms, promote functional recovery and allow for longer maintenance at home [1,2]. The GDH typically provides multidisciplinary assessment and rehabilitation in an ambulatory setting and has a pivotal position between hospital and home-based services[3].

Concern has been previously expressed that evidence for effectiveness is equivocal and that day hospital care is expensive[4]. Studies have also shown that the benefits of GDH rehabilitation outweighed its costs and is less costly compared to an equivalent period of in-patient rehabilitation or un-structured out-patient therapy[5]. However, a meta-analysis reviewing 12 clinical trials of GDH concluded that there was no clear advantage over other forms of care, but that patients attending GDH tended to have better outcomes than those receiving no comprehensive care[3].

The diversity of patients attending GDH and lack of standardized procedures may explain the observed discrepancies. We hypothesized that results could be improved if the right outcome measures and patients are selected as suggested by Hoe[2]. Furthermore, candidates for rehabilitation programs should be selected considering the real potential for improvement. It is important to identify older patients who will benefit most from post acute rehabilitative hospital admissions[6]. Targeting is essential to maximize health gains for patients in a system with limited resources.

The objectives of this study were thus: 1) to evaluate the effect of attending GDH on several measures of physical performance, functional capacity and quality of life; 2) define the characteristics of those who substantially improved and 3) identify factors best predictive of improvement.

## Methods

### Geriatric Day Hospital Setting

This retrospective cohort study was conducted at the Geriatric Day Hospital of the McGill University Health Center - Royal Victoria Hospital site (MUHC-RVH). This GDH serves a defined geographic region, which includes approximately 200,000 people in the downtown area of Montreal, Quebec. Our GDH is an outpatient interdisciplinary service that provides evaluation and/or rehabilitation to the frail elderly who are at least 65 years old and living in the community. Admission criteria for the GDH include patients with recent functional decline, multiple co-morbidities, having adequate physical endurance and cognitive capacity to tolerate a full-day rehabilitation program, motivation to participate, and the need for a multi-disciplinary team approach to care. The GDH staff consisted of a team coordinator, a geriatrician, two nurses, a physiotherapist, an occupational therapist and a patient attendant.

The study design was an inception cohort where data were extracted from records kept in a GDH clinical database (FileMaker Pro version 4.1, FileMaker Inc, Santa Clara, CA, USA) from April 2002 to March 2007. These data include: patient demographics, and results of standardized measurement tools used by the GDH professionals, done on admission and at discharge.

A patient typically attends the GDH twice a week from 9 AM to 3 PM. The GDH interventions included activities to achieve maximal functional independence in mobility, to prevent or correct disability and to maintain health. The patient's length of stay and discharge is individually determined by the setting of functional and measureable goals and their attainment or lack of progress.

### Patients and Variables Measured

The assessment tools used and recorded routinely include: Barthel Index for basic activities of daily living (ADL) [7]; Older Americans Resources and Services (OARS) for instrumental activities of daily living (IADL) [8]; Folstein Mini Mental Status Exam (MMSE) for cognitive function (only on admission) [9]; Timed Up & Go (TUG) for general mobility and locomotor performance [10]; 6-minute walk test (6 MWT) [11]; Gait speed [12]; Berg Balance Scale [13]; Grip strength [14] and the European Quality of life - 5 Dimensions (EQol5D) for perceived health status [15]. Visit dates and number of visits were also collected. Patients who were admitted to the rehabilitation program and were seen for 5 visits or more were included. Only those patients who had a complete data set on both admission and at discharge were used for the predictive component of the study. The study protocol was ethically approved by the Institutional Review Board of the McGill University Health Centre via the office of the Director of Professional Services.

### Definitions of Improvement

Improvement of functional performance measures from admission to discharge was defined as: Barthel score increase of 5 points or greater [16]; OARS increase of 1 point or more [17]; TUG decrease of 3 seconds or more [18]; Gait speed increase of 0.2 m/s or more [19]; 6 MWT increase of 30 meters or more [20]; Berg score increase of 3 points or more [13]; Grip strength increase of 2 kg or more [21]; and an EQol5 D score increase of 10 points or more [22]. These improvement thresholds were based on the published psychometric properties of the above functional measures. Since uncertainty remains around the value of clinical improvement in one single test, we created the definition of the attainment of "successfully improved" if there was improvement in at least 3 out of the 6 tests listed above (excluding grip strength and EQol5 D measures). A hospital chart review was done on a random ten percent sample to extract medical diagnostic information to generate a Charlson Comorbidity Index [23] and the number of concurrent medications as a proxy measure of "medical severity". To validate data integrity we compared the test results documentation in this sample of patient charts against the entries in the electronic clinical database and found no errors.

### Statistical Analysis

Descriptive analyses were compiled using proportions, means, standard deviations and range, as appropriate. Groups were compared using 95% confidence intervals based on the binomial or normal distributions, for proportions and means, respectively. Logistic regression was used on all nine measures, alone or in combinations to estimate the effects of possible predictors of rehabilitation success. The Bayesian Information Criterion (BIC) was used to select the optimal model for making future predictions of rehabilitation success [24]. Statistical analyses were performed using SAS (version 9; Statistical Analyses Software, SAS Institute, Cary, NC, USA) and R software packages (version 2.7.2; R Foundation for Statistical Computing, Vienna, Austria, 2009).

## Results

A total of 335 patients (218 females, 117 males) who attended GDH were in the rehabilitation program. Data for a complete set of tests done on admission and at discharge were found in 233(70%) patients. Patient characteristics and results of tests on admission are shown in Table 1. The mean age was 81.6 (SD = 6.9) years, range 58 to 98 years. Patients were predominantly female (65%), had a mean MMSE score of 26.7 (SD3.4), and participated in 24.2 (SD10.8) visits. The chart review data on a random 10% sample of patients yielded an average Charlson Comorbidity Index of 1.90, and an average medications count of 9.3. Results of the GDH intervention (Table 2) showed that rehabilitation had a significant positive effect. Approximately 45% of the patients improved in Barthel, OARS, Berg, Grip strength and EQol5 D tests after the rehabilitation period. Only 21% of patients showed improvement in their gait speed whereas in 6 MWT and TUG tests, 59% and 63% of patients improved, respectively. Significant correlations of variables on admission with changes in different tests were observed mainly for the test itself (data not shown). Thus, for Barthel r = -0.47 (CI: -0.59 - -0.43), for TUG, r = -0.57 (CI: -0.65 - -0.49), for 6 MWT, r = -0.33 (CI: -0.41 - -0.26) and for EQol5 D, r = -0.54 (CI: -0.65 - -0.39). Not surprisingly, gait speed correlated with changes in TUG r = 0.34 (CI: 0.27 - 0.41). Table 3 shows the characteristics of patients who achieved "successful improvement" criteria compared to those who did not. "Successful improvement" was attained in 58% (134/233) of patients. No significant differences were observed for age, gender, number of visits and baseline MMSE between patients who achieved "successful improvement" and those who did not. Patients who achieved "successful improvement" had lower admission performance scores in Barthel, OARS, TUG, gait speed and 6 MWT, with a trend for lower scores on the Berg test and EQol5 D. The logistic regression analysis generated a total of 512 models, and the best predictive model of "successful improvement" according to the BIC was the 6 MWT alone, OR: 0.994 (95% CI: 0.990 - 0.997) per meter of distance walked. Thus at admission, for each extra 20 meters walked on the 6 MWT (*i.e*. the patient is starting at a better functional level), that individual had an approximate 12% decrease (*i.e*. worse chance) in attaining "successful improvement".

**Table 1:** Patients' characteristics at admission

Variable	Mean (SD)	Range^2 ^(n)
Age (y)	81.6 (6.9)	58-98 (335)

Females	65%	

Medications (n)^1^	9.3	2-20 (34)

Charlson Co-morbidity Index^1^	1.90	0-7 (34)

Visits	24.2 (10.8)	5-85 (335)

Mini-Mental State Examination (on/30)	26.7 (3.4)	9-30 (304)

Barthel (on/100)	87.3 (13.0)	25-100 (311)

Older American Resources and Services (on/14)	8.1 (2.8)	2-14 (310)

Timed Up and Go (s)	27.4 (14.4)	0-93 (302)

Gait Speed (m/s)	0.6 (0.2)	0.1-1.0 (304)

6-Minute Walk Test (m)	157 (81)	10-360 (303)

Berg Balance Score (on/56)	39.1 (9.2)	8-55 (302)

Handgrip Right (kg)	13.8 (8.6)	0-63 (284)

Handgrip Left (kg)	12.9 (7.7)	0-53 (274)

European Quality of Life - 5 Dimensions on/100)	62.0 (16.7)	20-100 (141)

^1^Based on a random 10% selection of patient charts.

^2^Values of zero for functional measures mean the patient was unable to do the test

**Table 2:** Changes in test performance and proportion with significant improvement

	n	Admission Mean (SD)	Discharge Mean (SD)	Differences (95% CI)	Significant improve (%)
Barthel	251	87.7 (12.2)	91.4 (10.9)	3.68 (2.83, 4.55)	46.0

OARS	250	8.18 (2.8)	8.79 (2.8)	0.61 (0.43, 0.78)	47.0

Timed Up and Go	254	26.5 (13)	21.0 (10.8)	-5.52 (-4.46,-6.59)	62.7

Gait Speed	267	0.56 (0.20)	0.64 (0.21)	0.078 (0.060, 0.097)	21.3

6-Minute Walk Test	266	155.7 (79.6)	199.6 (81.7)	43.84 (36.87, 50.8)	58.8

Berg Balance Score	261	39.29 (8.6)	43.2 (8.5)	3.91 (3.25, 4.56)	47.7

Handgrip Right	141	12.3 (7.4)	14.6 (7.8)	2.25 (1.68, 2.82)	43.3

Handgrip Left	125	11.7 (7.3)	13.4 (7.7)	1.77 (1.27, 2.27)	39.2

EQol5D	104	63.4 (17.2)	72.5 (15.9)	9.06 (5.83, 12.28)	46.7

**Table 3:** Patient's characteristics on admission associated with successful improvement (significant improvement on 3 tests or more)

Variables	Successful Mean (SD)	Unsuccessful Mean (SD)	Differences (95% CI)
N	134	99	

Age	81.6 (7.1)	82.1 (6.6)	-0.51 (-0.047, 0.20)

Sex; F:M ratio	1.69	1.62	0.078 (-2.31, 1.30)

MMSE	26.9 (3.0)	27.0 (3.5)	-0.13 (-0.97, 0.71)

Barthel	87.4 (11.1)	90.9 (9.5)	-3.57 (-6.30, -0.84)

OARS	8.0 (2.6)	8.7 (2.7)	-0.71 (-1.41, -0.012)

Timed Up and Go	27.9 (12.5)	23.9 (11.7)	4.01 (0.83, 7.19)

Gait Speed	0.53 (0.18)	0.61 (0.20)	-0.081 (-0.13, -0.030)

6-Minute Walk Test	141.5 (74.8)	178.6 (80.3)	-37.1 (-57.27, -16.96)

Berg Balance Score	38.7 (8.3)	40.8 (8.1)	-2.08 (-4.23, 0.058)

EQol5D	60.0 (14.4)	65.4 (19.7)	-5.38 (-11.64, 0.86)

## Discussion

This study presents data on a large series of GDH patients. We found a positive effect of the GDH rehabilitation program on our patient population from admission to discharge when we considered multiple functional measures. This positive effect has previously been shown in other GDH programs when considering only ADL and/or TUG measures [6,25-27]. Even though individual functional performance measures such as gait speed, TUG and 6 MWT have proven clinical relevance, the variability of associations with successful rehabilitation outcomes make them an uncertain choice for predicting those who do well. Patients who attained "successful improvement" by our definition (improvement in 3 or more tests of physical performance) and therefore rehabilitation success had lower scores on admission for all measures except for the MMSE. Similar results have been reported by Malone, as patients who scored below the median at admission showed a trend towards improvement [7]. These findings can be explained in part by the fact that it is harder to improve further when the measure score is already high on admission (*i.e*. ceiling effect) and perhaps in part by a regression to the mean. Conversely, patients with very low functional measure scores on admission may have poor rehabilitation potential [28]. Another confounder is that even though the psychometric properties of each of the functional measures have well-defined thresholds detecting significant change, we have no way of determining if improvement in one of the tests is easier to attain than in another. Age showed a weak negative correlation and cognitive function a weak positive correlation with Grip strength. This observation is consistent with the variability reported [27] showing a negative effect of age [6] and lower cognitive function on response to GDH rehabilitation [7,25]. Despite low functional scores, many patients have the potential to improve. This point is certainly applicable to the home rehabilitation patient, who would more likely have more functional impairments compared to our GDH patients.

Several factors reported in the literature are potentially predictive of poor functional outcomes following a course of in-patient geriatric rehabilitation [29]. Logistic regression analysis using the BIC to identify predictive models of successful GDH rehabilitation showed that the 6 MWT alone was significant, OR = 0.994/metre. In other words for each 100 metres distance walked on admission, the OR = 0.55 (95% CI: 0.37 - 0.74). The interpretation of this result means that when comparing two patients on admission, the patient who walked 100 m further would on average have a 45% reduction in their probability of attaining "successful improvement". The small sample size makes it difficult to apply sensitivity analysis to determine a cutoff value for the 6 MWT that could predict a patient's chance of benefiting from rehabilitation. Of interest, when the 102 participants excluded from the predictive model due to lack of discharge data were compared respectively to the rest of the patients for the variables listed on Table 1, three tests were statistically different: Barthel mean (85.45 *vs*. 88.76 p < 0.001), TUG mean (29.39 *vs*. 26.17 p < 0.001) and Berg mean (37.37 *vs*. 39.60 p < 0.001), albeit of minimal clinical significance. Our analysis generated all 512 possible models, considering the nine functional measures alone or in combinations. Nevertheless, we were unable to find a set of reliable predictors for the attainment of "successful improvement". This failure is similar to other studies [7] and heterogeneity (case mix) in the GDH population may be partly responsible for this difficulty [3,26], along with the small sample size compared to what is typically needed for predictive model building.

Medical diagnosis (co-morbidities) and number of medications have also been considered as predictors. Although we extracted information on only a small sample of charts on our patients, which is insufficient to interpret our results, other studies have reported that the number of medical diagnoses or medications have not been shown to be predictive of outcome in the GDH setting [26]. In our GDH population, the rehabilitation outcome is more directly related to the functional measures on admission, regardless of a high average burden of disease and medication count. For the present time and considering that GDH patients have diverse diagnoses, function capacities and variable cognitive abilities, rehabilitation teams must develop an individual program on a case-by-case basis. The goals that are set must be patient-specific, functional, measureable and consider the individual patient's limitations and potential [26].

This study has the limitations inherent in a retrospective analysis. Missing data contributed to a decreased sensitivity of our prediction models. The lack of formal data on patient motivation or the presence of depression, which has been shown to predict rehabilitation outcome [30] also impairs our interpretations. However, the GDH team informally assesses these psychological factors during their regular team meetings, which monitors each patient's rehabilitation progress and determines time for discharge. The long term impact on socio-medical measures at 6 or 12 months would be an important outcome to investigate the durability of a successful rehabilitation episode. Recording of International Classification of Function (ICF) participation level may be able to identify patient profiles that would further enhance targeting of patients to the GDH rehabilitation program. This would be a topic of a separate prospective study.

## Conclusions

The GDH ambulatory rehabilitation program had positive effects on most measures of physical function capacity in frail elderly patients. No single test or combination of tests was sufficient to assist in accurate patient targeting for rehabilitation in our GDH setting.

Whether our results can be extrapolated to a home treatment program is uncertain, but the supporting evidence that poorly functioning individuals have the most to gain from a rehabilitation program is promising. Our observations emphasize the need for a prospective study with innovative and appropriate outcome measures for GDH [2,25] with the aim of developing tools to better target those frail elderly patients who will benefit most from rehabilitation. The addition of items pertaining to ICF participation may also shift the focus from purely measured functional outcomes to a set of patient reported outcomes.

## Competing interests

The authors declare that they have no competing interests.

## Authors' contributions

SP, WC, AT and SC participated in the study design, data acquisition, interpretation of data and manuscript preparation.

LJ did the statistical analysis, interpretation of data and participated in the manuscript preparation.

AH and JM participated in the study design, interpretation of data and manuscript preparation and review.

## Pre-publication history

The pre-publication history for this paper can be accessed here:

http://www.biomedcentral.com/1471-2318/10/82/prepub
